# A comparative study of different standards of iron supplementation in pregnant women with iron deficiency in plateau area: A retrospective cohort study from a single center in China

**DOI:** 10.1097/MD.0000000000046327

**Published:** 2026-05-12

**Authors:** Yang Yao, Lanxu Sun, Xin Zuo, Zenglin Yang, Yan Yang, Jing Luo

**Affiliations:** a Gynecology and Obstetrics, Kunming First People’s Hospital, Kunming City, Yunnan Province, China.

**Keywords:** iron deficiency anemia during pregnancy, iron deficiency during pregnancy, plateau section, serum ferritin

## Abstract

This study investigates the effect of starting iron supplementation with serum ferritin (SF) < 30 μg/L in pregnant women with iron deficiency at Plateau area. This retrospective cohort study included 701 pregnant women with iron deficiency in early pregnancy who delivered in the First People’s Hospital of Kunming, China, from January 1, 2022 to July 31, 2023. Iron deficiency was defined as hemoglobin (Hb) ≥ 110 g/L and SF < 30 μg/L during pregnancy. The effect and pregnancy outcome of iron supplementation with SF < 30 μg/L as the standard and Hb < 120 g/L as the standard were compared by statistical methods. Among 701 cases of iron deficiency in the first trimester, 342 cases were given SF < 30 μg/L as the standard of preventive iron supplementation (Group A), and 359 cases were given Hb < 120 g/L as the standard of preventive iron supplementation (Group B). This study found that the Group A had a statistically significant difference in the incidence of anemia and iron deficiency before delivery compared with the Group B (*P* < .001), and it was found that the Group A had a higher Hb level before delivery (β: 6.693, 95% confidence interval: 5.691–7.696) and those with pre-delivery SF ≥ 30 μg/L had a higher rate of recovery and a faster recovery time (hazard ratio: 10.77, *P* < .0001). This relationship remained stable after multivariate adjustment using linear regression analysis, and Kaplan–Meier plots of recovery of SF ≥ 30 μg/L during 280 days of gestation also showed significant differences between the 2 groups. In addition, In addition, the incidence of postpartum hemorrhage was significantly lower in the Group A (odds ratio: 0.374, 95% confidence interval: 0.155–0.901), and this association remained stable after adjusting for confounding factors by multivariate binary logistic regression analysis. Other pregnancy outcomes were not statistically significant. In plateau area, pregnant women with iron deficiency who start preventive iron supplementation with SF < 30 μg/L can reduce the incidence of iron deficiency and anemia before delivery compared with those who start preventive iron supplementation with Hb < 120 g/L, improve the level of Hb before delivery and the recovery rate and recovery time of SF ≥ 30 μg/L, and reduce the incidence of postpartum hemorrhage.

## 1. Introduction

Anemia, the most prevalent nutritional deficiency worldwide, is a common complication during pregnancy. The World Health Organization defines anemia during pregnancy as having hemoglobin (Hb) levels below 110 g/L.^[1]^ Surveys from 2019 show a 36% prevalence of anemia during pregnancy, posing a significant risk to expectant mothers.^[2]^ Particularly in low- and middle-income countries, anemia remains a leading cause of maternal mortality and adverse pregnancy outcomes.^[3]^ Iron deficiency (ID) is the primary cause of anemia in pregnancy, accounting for 95% of cases.^[4]^ Maternal iron deficiency is identified as having serum ferritin (SF) levels below 30 μg/L.^[5,6]^ Research indicates that the incidence of iron deficiency anemia (IDA) and ID during pregnancy can be as high as 20.4% and 80%, respectively.^[7]^ Various studies have highlighted the potential for adverse short- and long-term consequences for both mothers and infants resulting from IDA and ID during pregnancy.

The World Health Organization recommends daily iron and folic acid supplementation during pregnancy to prevent adverse outcomes like anemia, preterm birth, and small for gestational age. However, there is currently no consensus on the significance, timing, and dosage of iron supplementation for pregnant women, both domestically and internationally.^[8–10]^ Previous studies have shown that the incidence of ID and IDA varies among pregnant women in different regions due to differences in diet, income levels, and living habits.^[11]^ In plateau areas, which are located above 1000 m above sea level where oxygen levels are low, the human body’s hemoglobin levels are often higher to acclimate to the environment. Additionally, factors such as religious beliefs, cultural practices, low education levels, and economic disparities contribute to higher rates of anemia during pregnancy.^[12]^ Using the WHO diagnostic criteria for anemia during pregnancy (Hb < 110 g/L) in plateau areas may lead to overlooking pregnant women who do not appear anemic but may have developed ID, thus missing the opportunity to prevent IDA during pregnancy. Current clinical studies have demonstrated that hemoglobin concentration has a U-shaped relationship with adverse pregnancy outcomes.^[13]^ Hemoglobin levels higher than 130 g/L may increase the incidence of gestational diabetes mellitus, preeclampsia, premature delivery, and low birth weight.^[14]^

Therefore, in this study, pregnant women with ID were selected as subjects to improve the safety and accuracy of preventive iron supplementation. At the same time, SF with higher sensitivity was selected as the detection index. SF, an acute phase protein produced by the liver, serves as a key indicator of iron storage in the human body and is fundamental for diagnosing ID.^[15]^ Numerous domestic and international guidelines advocate for SF as the diagnostic marker for IDA, suggesting that SF levels below 30 μg/L during pregnancy should prompt the initiation of iron supplementation for prevention.^[5,6,15]^

However, there is currently a lack of research on preventive measures for IDA and ID in pregnant women living in plateau area. Therefore, we will conduct a retrospective cohort study aimed at investigating the effect of SF or Hb as standard preventive iron supplementation in pregnant women with ID in a plateau area. In order to prevent ID and IDA during pregnancy in plateau area more effectively, so as to improve pregnancy complications and adverse outcomes.

## 2. Materials and methods

### 2.1. Study approval and registration

A retrospective cohort study was conducted in the obstetrics department of the First People’s Hospital of Kunming, Yunnan Province, China. This general tertiary hospital, located in a plateau area at 1891 m above sea level, handles over 4000 deliveries annually. The study adhered to the Declaration of Helsinki principles and Chinese statutory regulations, receiving approval from the Ethics Committee of the First People’s Hospital of Kunming in September 2023 (Ethics approval No. YLS2023-70). Data collection commenced on October 1, 2023. Patients received information about studies being conducted on their biological samples and signed written informed consent to participate in the study.

### 2.2. Inclusion and exclusion criteria

This retrospective study included 6583 pregnant women who were hospitalized in the Department of Obstetrics, Kunming First People’s Hospital from January 1, 2022, to July 31, 2023. Among them, 701 women met the inclusion and exclusion criteria and completed the entire treatment process of preventive iron supplementation and blood sample collection.

The inclusion criteria were as follows: singleton pregnant women; pregnant women who delivered in the Department of Obstetrics of Kunming First People’s Hospital from January 1, 2022 to July 31, 2023; and pregnant women living in Kunming for more than 1 year;

Exclusion criteria were as follows: pregnant women with Hb <110 g/L or SF ≥ 30 μg/L in the first trimester were excluded (The subjects in this study were selected from the ID population during pregnancy. Subjects with anemia Hb < 110 g/L during pregnancy and normal subjects with SF ≥ 30 μg/L were excluded); pregnancy complicated with heart disease, chronic hypertension, type 1/type 2 diabetes mellitus were excluded; special types of anemia excluding iron deficiency anemia (such as thalassemia, aplastic anemia, megaloblastic anemia and other anemia caused by non-iron deficiency); pregnant women who refused to take iron or interrupted taking iron were excluded; C-reactive protein > 5 mg/L; pregnant women with incomplete data were excluded; and pregnant women with twin or multiple births.

### 2.3. Grouping and iron supplementation criteria

According to the simple random method of 1:1, the attending physician divided the subjects who met the inclusion and exclusion criteria into 2 groups (using Excel to register the patient information, selected into group A according to the odd number, and selected into group B according to the even number):

Group A: Oral iron supplementation should be initiated in pregnant women with Hb ≥ 110 g/L and SF < 30 μg/L in the first trimester, when SF < 30 μg/L.

Group B: Oral iron supplementation should be initiated in pregnant women with Hb ≥ 110 g/L and SF < 30 μg/L in the first trimester, when Hb < 120 g/L.

According to the recent guidelines at home and abroad,^[6,15–17]^ preventive iron supplementation is more acceptable for pregnant women with high risk factors of ID during pregnancy or SF < 30 μg/L. The recommended intake of elemental iron is 27–80 mg/d. Because hepcidin levels vary between day and night, the concentration is the lowest in the morning,^[18]^ and it is recommended to take oral elemental iron in the morning on an empty stomach. Commonly used oral iron agents include: inorganic iron (ferrous sulfate, etc), organic iron (polysaccharide iron complex, ferric protein succinate, ferrous fumarate, ferrous succinate, and ferrous gluconate, etc).^[15]^ Therefore, in this study, we chose ferrous succinate tablets 80 mg/d, taken orally in the morning on an empty stomach.

### 2.4. Diagnostic criteria and definitions

The following are the diagnostic criteria for the diseases covered in the article:

1. Definition of anemia during pregnancy: hemoglobin < 110 g/L.^[4]^
2. Definition of ID during pregnancy: SF < 30 μg/L.^[15]^
3. Definition of gestational hypertension: hypertension occurred after 20 weeks of pregnancy, systolic blood pressure ≥ 140 mm Hg and/or diastolic blood pressure ≥ 90%, returned to normal within 12 weeks after delivery, and could not be diagnosed until postpartum.^[4]^
4. GDM definition: all pregnant women who had not been diagnosed as GDM, the OGTT diagnostic criteria at 24 to 28 weeks of gestation and the first visit after 28 weeks of gestation were as follows: fasting, 1-hour and 2-hour blood glucose levels below 5.1 mmol/L, 10.0 mmol/L and 8.5 mmol/L, respectively. GDM was diagnosed when any point of blood glucose value reached or exceeded the above criteria.^[4]^
5. Definition of postpartum hemorrhage: postpartum hemorrhage is a serious childbirth complication, which refers to the amount of bleeding ≥ 500 mL in vaginal delivery and ≥ 1000 mL in cesarean delivery within 24 hours after the delivery of the fetus.^[4]^
6. Definition of neonatal asphyxia: it refers to the inability of newborns to carry out normal spontaneous breathing after birth, which is one of the main causes of neonatal death and intellectual disability. Apgar score ≤ 7 is diagnosed as neonatal asphyxia.^[4]^
7. Postpartum transfusion: transfusion within 7 days after delivery.
8. Preterm birth: the term “preterm birth” refers to the birth at 28 but <37 weeks of gestation, and the newborn born at this time is called premature.^[4]^
9. Low birth weight infants: newborns whose birth weight is <2500 grams after term are called term low birth weight infants.^[4]^

### 2.5. Demographic, anthropometric characteristics, pregnancy complications, and pregnancy outcomes

We recorded clinical data, pregnancy complications and pregnancy outcomes of all participants, including age, BMI before delivery, number of pregnancies, and pre-pregnancy comorbidities (e.g., chronic hypertension, type 1/type 2 diabetes, heart disease, etc). And pregnancy complications and adverse pregnancy outcomes, including gestational diabetes mellitus, gestational hypertension, postpartum hemorrhage, postpartum blood transfusion, neonatal asphyxia, preterm birth, and low birth weight infants.

### 2.6. Statistics of biochemical indicators

Blood samples were obtained for the following laboratory tests, available in the hospital’s electronic medical record system: blood cellanalysis, C-reactive protein, and serum ferritin, during the first trimester and every 4 weeks from the first trimester and up to 72 hours before delivery.

### 2.7. Outcomes of the study

Primary outcome: The study compared the incidence of pregnancy complications (gestational hypertension, gestational diabetes mellitus) and adverse pregnancy outcomes (postpartum hemorrhage, postpartum blood transfusion, neonatal asphyxia, preterm birth, and low birth weight) between the 2 groups. Secondary outcome: It examined the effect of iron supplementation by measuring Hb levels at 3 days before delivery and the recovery rate of serum ferritin ≥ 30 μg/L at 280 days of gestation. The recovery rate of serum ferritin ≥ 30 μg/L was monitored throughout the 280-day gestation period through blood cell analysis, C-reactive protein, and serum ferritin measurements every 4 weeks, with Kaplan–Meier curves used to compare the rate of recovery.

### 2.8. Statistical methods

For clinical and biological data, Microsoft Excel software was used for data recording and analyses were performed using SPSS v27.0. The quantitative data are described as median values [Interquartile] and categorial variables as frequencies and percentages. For quantitative variables, the non-parametric Kruskal–Wallis tests were used for comparison between groups. For qualitative variables, chi-square tests were used to compare groups or Fisher tests when expected values were below 5. Correlations between variables were calculated using the non-parametric Spearman rank-order test. *P* values < .05 were considered significant. Univariate and multivariate linear regression analysis were used to analyze the effect of preventive iron supplementation: hemoglobin level 3 days before delivery. Kaplan–Meier curves were used to compare the rate of recovery of serum ferritin ≥ 30 μg/L at 280 days of gestation. Univariate and multivariate binary logistic regression models were used to compare pregnancy complications and pregnancy outcomes to reduce confounding factors.

## 3. Results

### 3.1. Flowchart for patient selection

**Figure 1.**
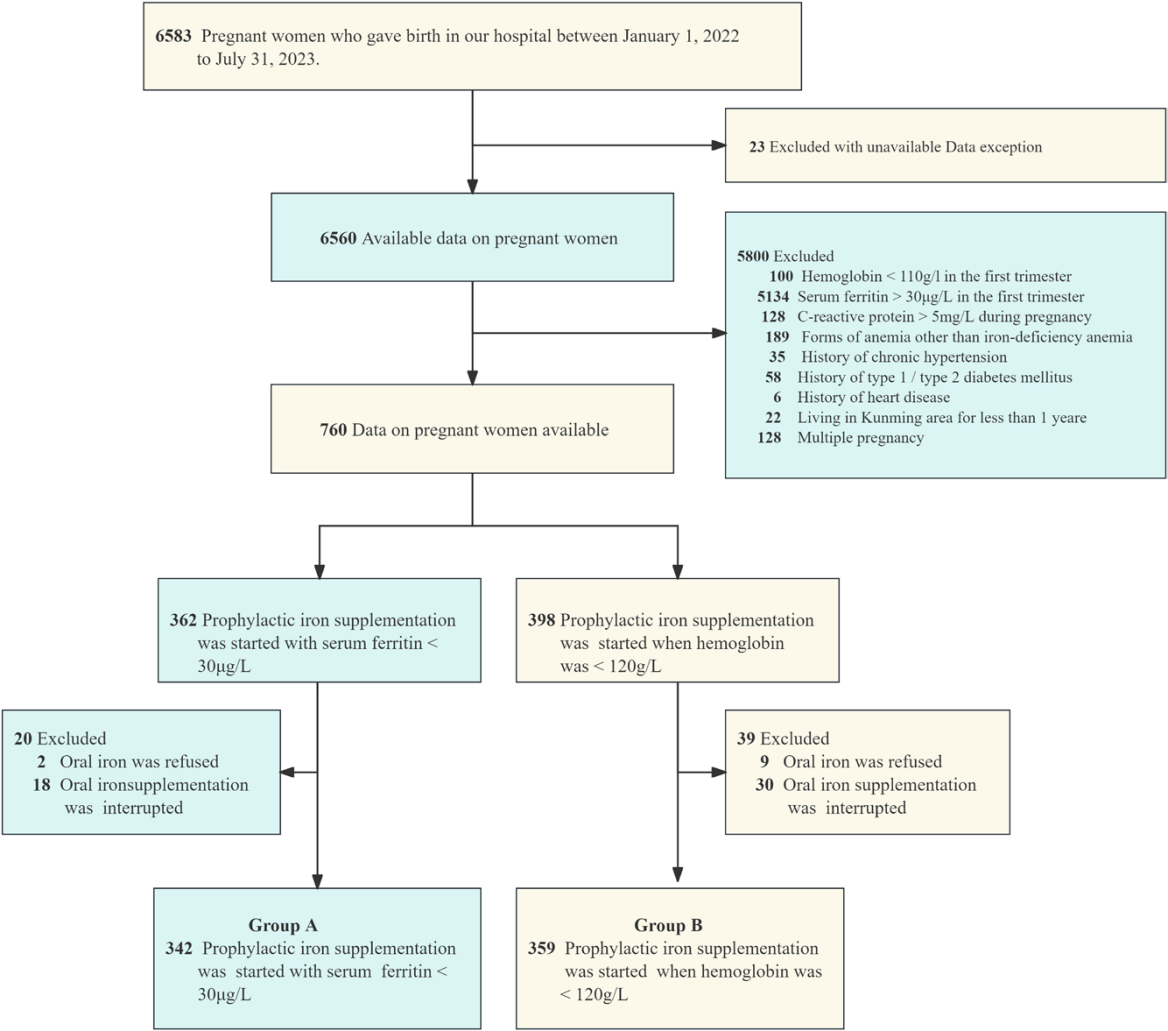
Flow chart of patient selection.

This retrospective study included 6583 pregnant women who delivered in the Department of Obstetrics, Kunming First People’s Hospital from January 1, 2022 to July 31, 2023. Exclusion data could not be obtained for 23 cases, leaving 6560 cases. Then 5800 pregnant women were excluded, including Hb < 110 g/L in the first trimester (n = 100), SF ≥ 30 μg/L in the first trimester (n = 5134), other special types of anemia (n = 189), history of chronic hypertension (n = 35), type 1/type 2 diabetes (n = 58), and pregnancy complicated with heart disease (n = 6), duration of residence in Kunming < 1 year (n = 22), twin and multiple pregnancy (n = 128). Remaining patients n = 760 (e.g., Fig. 1). They were divided into 2 groups by simple random method and treated with different standards of preventive iron supplementation. Pregnant women who interrupted iron supplementation (n = 48) and who refused iron supplementation (n = 11) were excluded. Finally, 701 subjects were left. Group A, n = 342: Oral iron supplementation should be initiated in pregnant women with Hb ≥ 110 g/L and SF < 30 μg/L in the first trimester, when SF < 30 μg/L. Group B, n = 359: Oral iron supplementation should be initiated in pregnant women with Hb ≥ 110 g/L and SF < 30 μg/L in the first trimester, when Hb < 120 g/L.

### 3.2. Demographic and clinical characteristics of the patients

The study involved 701 women with an average age of 30 years, a pre-delivery BMI of 24.8 kg/m^2^, 3 pregnancies, a first-trimester Hb level of 130 g/L, and SF of 24.1 μg/L. Demographic and clinical characteristics of all subjects, as well as a comparison between the 2 groups, are presented in Table 1. There were no significant differences in age, pre-delivery BMI, number of pregnancies, Hb level in early pregnancy, SF level in early pregnancy, and gestational age at delivery between the 2 groups. Group A initiated iron supplementation approximately 14.5 weeks earlier than group B (*P* < .001).

**Table 1:** Maternal clinical characteristics.

Different standards of iron supplementation	Total	Group A	Group B	*P* value
Maternal characteristics (n)	n = 701	n = 342	n = 359	
Median age, years	30 (27–33)	30 (27–33)	30 (27–33)	.868
Predelivery BMI, kg/m²	24.8 (23.7–25.8)	24.75 (23.7–25.7)	24.8 (23.7–25.8)	.555
Number of pregnancies	3 (2–4)	3 (2–4)	3 (2–4)	.979
Gestational age at delivery, weeks	38 (38–39)	38 (38–39)	38 (38–39)	.847
Serum ferritin in early pregnancy, μg/L	24.1 (21.1–27.0)	24.3 (21.2–27.2)	24 (20.8–26.8)	.457
Hemoglobin in early pregnancy, g/L	130 (124–135)	130 (124–134)	130 (124–135)	.627
Gestational age of iron supplementation, weeks	20 (10–24)	9.5 (8–12)	24 (20–24)	**<.001**

Group A: Oral iron supplementation should be initiated in pregnant women with Hb ≥ 110 g/L and SF < 30 μg/L in the first trimester, when SF < 30 μg/L.

Group B: Oral iron supplementation should be initiated in pregnant women with Hb ≥ 110 g/L and SF < 30 μg/L in the first trimester, when Hb < 120 g/L.

*P* < .05 is of significance.

### 3.3. Effect of iron supplementation between the 2 groups

**Figure 2.**
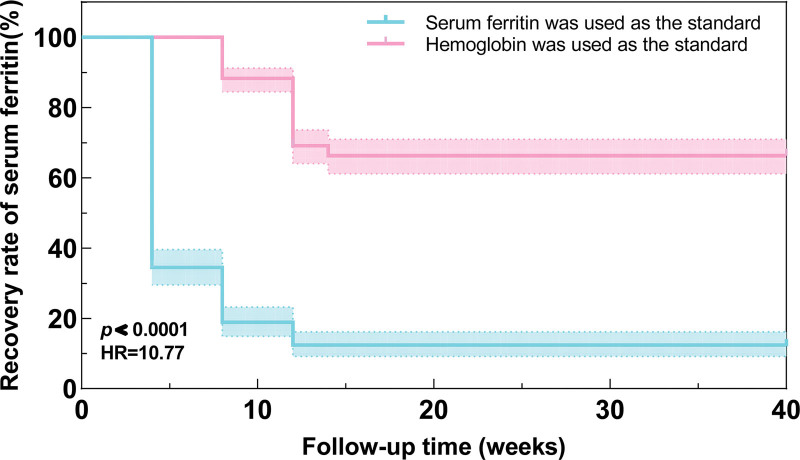
Kaplan–Meier method was used to analyze the recovery of SF ≥ 30 μg/L during 280 days of gestation.

Table2 observed that among 701 pregnant women in this study, the incidence of IDA and ID 3 days before delivery was 15.7% and 39.9%, respectively. Compared with group B, the incidence of IDA 3 days before delivery in group A (A: 20 (5.8%), B: 90 (25.1%)) and the incidence of ID 3 days before delivery in group A (A: 42 (12.3%), B: 238 (66.3%)) were significantly decreased (*P* < .001). Meanwhile, Table 2 observed that there were significant differences in Hb level and SF ≥ 30 μg/L recovery rate between the 2 groups 3 days before delivery (*P* < .001). Patients in group A had higher pre-delivery Hb levels (A: 123 (120–128), B: 117 (110–121)), and the recovery rate of SF ≥ 30 μg/L within 3 days before delivery was significantly higher (A: 300 (87.7%), B: 121 (33.7%)). We used the Kaplan–Meier method to analyze the difference in the recovery of SF ≥ 30μg/L during the 280 days of gestation between the 2 groups. The Kaplan–Meier plot shows A significant difference between the 2 groups (see Fig. 2). Group A had a faster recovery time and a higher recovery rate of SF than group B (HR: 10.77, *P* < .0001). Additionally, a linear regression analysis of the Hb levels before delivery in both groups (Table 3) demonstrated a noteworthy increase in Hb levels before delivery in group A (β: 6.693, 95%CI: 5.691–7.696), even after adjusting for factors such as age, number of pregnancies, BMI before delivery, Hb levels in early pregnancy, and SF levels (β: 6.556, 95%CI: 5.865–7.246). Notably, the adjustment for confounding variables did not alter the relationship between the 2 groups.

**Table 2:** The effect of iron supplementation before delivery was compared between the two groups.

Different standards of iron supplementation	Total	Group A	Group B	*P* value
Maternal characteristics (n)	n = 701	n = 342	n = 359	
Pregnant women with IDA 3 days before delivery, n	110 (15.7%)	20 (5.8%)	90 (25.1%)	**<.001**
Pregnant women with ID 3 days before delivery, n	280 (39.9%)	42 (12.3%)	238 (66.3%)	**<.001**
Hemoglobin in the third trimester, g/L	120 (115–125)	123 (120–128)	117 (110–121)	**<.001**
Recovery rate of SF ≥ 30 μg/L 3 days before delivery	421 (60.05%)	300 (87.7%)	121 (33.7%)	**<.001**

Group A: Oral iron supplementation should be initiated in pregnant women with Hb ≥ 110 g/L and SF < 30 μg/L in the first trimester, when SF < 30 μg/L.

Group B: Oral iron supplementation should be initiated in pregnant women with Hb ≥ 110g/L and SF < 30μg/L in the first trimester, when Hb < 120g/L.

IDA = iron deficiency anemia, ID = iron deficiency, SF = serum ferritin.

*P* < .05 is of significance.

**Table 3:** Linear regression analysis of preventive iron supplementation with SF < 30 μg/L as the standard and hemoglobin in the third trimester.

	Non-adjusted			Adjusted		
	β	*P*	95% CI	β	*P*	95% CI
Hemoglobin in the third trimester	6.693	<.001	5.691–7.696	6.556	<.001	5.865–7.246

Multivariate linear regression models were adjusted for: age, BMI, number of pregnancies, first-trimester hemoglobin, gestational hypertension, and gestational diabetes mellitus.

CI = confidence interval.

### 3.4. Pregnancy complications and adverse pregnancy outcomes were compared between the 2 groups

Table 4 displays the characteristics of pregnancy complications and adverse pregnancy outcomes in both groups. Group A had a notably lower incidence of postpartum hemorrhage compared to group B (*P* = .023). While there was no significant difference in postpartum blood transfusion between the groups (*P* = .067), the number of blood transfusions in group A was significantly lower than in group B (A: 1 (0.3%), B: 6 (1.7%)). No significant variances were observed in other pregnancy complications and adverse pregnancy outcomes between the 2 groups.

**Table 4:** The pregnancy complications and adverse outcomes of the 2 groups were compared.

Different standards of iron supplementation	Total	Group A	Group B	*P* value
Maternal characteristics (n)	n = 701	n = 342	n = 359	
Pregnancy complications				
Gestational diabetes	103 (14.69%)	48 (14%)	55 (15.3%)	.847
Pregnancy induced hypertension	36 (5.13)	17 (5.0%)	19 (5.3%)	.631
Adverse pregnancy outcomes				
Postpartum hemorrhage	26 (3.7%)	7 (2.0%)	19 (5.3%)	**.023**
Blood transfusion	7 (0.99%)	1 (0.3%)	6 (1.7%)	.067
Neonatal asphyxia	28 (3.99%)	13 (3.8%)	15 (4.2%)	.799
Infant of low-birth weight	49 (6.99%)	22 (6.4%)	27 (7.5%)	.572
Premature delivery	54 (7.7%)	26 (7.6%)	28 (7.8%)	.922

Group A: Oral iron supplementation should be initiated in pregnant women with Hb ≥ 110 g/L and SF < 30 μg/L in the first trimester, when SF < 30 μg/L.

Group B: Oral iron supplementation should be initiated in pregnant women with Hb ≥ 110 g/L and SF < 30 μg/L in the first trimester, when Hb < 120 g/L.

*P* < .05 is of significance.

Table 5 presents the multivariate logistic regression model of the association between initiation of iron supplementation with SF < 30 μg/L and postpartum hemorrhage in pregnant women with ID during pregnancy. Both unadjusted and multivariable adjusted models were utilized to ensure result stability. The selection of adjustment variables was guided by 3 criteria: Those that would alter the matched odds ratio by at least 10% when included in the model; variables with *P*-values < .05 in correlation analysis; variables identified as confounders based on existing literature and clinical judgment. Exclusion of variables with significant multicollinearity was performed. Tolerance values and variance inflation factor tests confirmed the absence of multicollinearity in the final model. Adjustment for confounding factors did not alter the association between iron supplementation and postpartum hemorrhage (odds ratio [OR]: 0.263, 95% confidence interval [CI]: 0.088–0.788), it is suggested that starting iron supplementation in ID pregnant women with SF < 30 μg/L may reduce the incidence of postpartum hemorrhage.

**Table 5:** Multivariate logistic regression model of the association between prophylactic iron supplementation and postpartum hemorrhage in pregnant women initiated with SF < 30 µg/L.

Model	*P*	OR	95% CI
Model 1	0.028	0.374	0.155–0.901
Model 2	0.029	0.374	0.155–0.901
Model 3	0.029	0.374	0.155–0.901
Model 4	0.017	0.263	0.088–0.788

*P* < .05 is of significance. Model 1: Crude model. Model 2: Adjust for age, BMI, number of pregnancies. Model 3: Adjust for Model 2 + Serum ferritin and hemoglobin in early pregnancy. Model 4: Adjust for Model 3 + gestational hypertension and gestational diabetes mellitus.

CI = confidence interval, OR = odds ratio.

## 4. Discussion

IDA is a prevalent condition during pregnancy globally and a key risk factor for adverse pregnancy outcomes.^[19]^ Maternal anemia rates are particularly high in plateau regions.^[20]^ This retrospective cohort study conducted at the First People’s Hospital of Kunming (1891 m altitude) revealed that 1.52% of pregnant women in the first trimester had IDA, while 13.1% had ID. This is similar to a Chinese survey, a 2018 survey of pregnant women in urban areas of China reported IDA prevalence of 1.96%, 8.40%, and 17.82% in the first, second, and third trimesters, respectively.^[21]^ But adjusting for altitude using the CDC method, the incidence of IDA in early pregnancy was 7.7%, significantly higher than that in plain areas.In this study, we observed that in pregnant women with ID, the initiation of iron supplementation with SF < 30 μg/L was significantly earlier in gestational weeks than that with Hb < 120 g/L as the standard, and it also reduces the incidence of ID and IDA 3 days before delivery in pregnant women with ID in the first trimester. It was found that there were statistically significant differences between the 2 groups in Hb level 3 days before delivery and the recovery rate of SF ≥ 30 μg/L 3 days before delivery. Linear regression analysis showed that the Hb level of ID pregnant women with SF < 30 μg/L was significantly increased before delivery, and the relationship remained stable after multivariate adjustment. Kaplan–Meier analysis was used to analyze the difference in the recovery of SF ≥ 30 μg/L between the 2 groups during 280 days of gestation. Kaplan–Meier curve showed that the recovery rate of SF ≥ 30 μg/L was higher and the recovery time was earlier in ID pregnant women who started iron supplementation with SF < 30 μg/L. Additionally, comparison of complications of pregnancy and pregnancy outcomes between the 2 groups showed that women who received iron supplementation with SF levels below 30 μg/L had a lower incidence of postpartum hemorrhage. Multivariate logistic regression analysis showed that the association still existed after adjusting for confounding factors such as age, BMI before delivery, number of pregnancies, Hb and SF levels in the first trimester. However, there were no statistically significant differences in other pregnancy complications and outcomes such as gestational hypertension, gestational diabetes mellitus, postpartum blood transfusion, neonatal asphyxia, preterm birth, and low birth weight.

At present, there is a lack of research on iron supplementation measures during pregnancy in Plateau areas. Compared with previous studies, this study observed that starting iron supplementation with SF < 30 μg/L in ID pregnant women in the first trimester could reduce the incidence of ID and IDA before delivery, and improve the recovery rate and recovery time of Hb level before delivery and SF ≥ 30 μg/L in ID pregnant women. These findings align with most studies conducted in low-altitude regions. A meta-analysis of Cochrane revealed that preventive iron supplementation could significantly decrease the risk of ID and IDA at term, And it also increased the level of hemoglobin at term and after delivery.^[8]^ This may be because pregnant women with SF < 30 μg/L start preventive iron supplementation earlier, and intervention in the early stage of iron depletion can more effectively improve hemoglobin and serum ferritin levels, thereby reducing the incidence of ID and IDA before delivery. At the same time, we also found that the incidence of postpartum hemorrhage in ID pregnant women with SF < 30 μg/L as the standard for preventive iron supplementation was significantly reduced, which may be due to the high correlation between postpartum hemorrhage and prenatal anemia. During the delivery of pregnant women with anemia, the uterus would be hypoxic, which would lead to the loss of uterine muscle tension and the failure to contract blood vessels, resulting in massive blood loss.^[22]^ The ID pregnant women with SF < 30 μg/L as the standard of preventive iron supplementation have a higher Hb level before delivery, and also reduce the occurrence of IDA before delivery, thereby reducing the incidence of postpartum hemorrhage. It is also possible that ID stimulates the expression of matrix metalloproteinases, and high expression of matrix metalloproteinases can reduce uterine contractions and induce postpartum hemorrhage.^[23]^ We also found that there were no significant differences in other pregnancy complications and pregnancy outcomes: gestational hypertension, gestational diabetes, postpartum blood transfusion, neonatal asphyxia, preterm birth and low birth weight. These findings are different from those of previous studies. The results of 0h et al^[24]^ and Abioye et al^[25]^ suggest that the use of multiple nutrient supplements containing iron is more beneficial to pregnancy outcomes such as preterm birth and low birth weight infants, and does not lead to any adverse outcomes. These differences may be due to the population with ID during pregnancy in the plateau region we selected, and more studies are needed to explore.

The highlight of this study is the careful selection of the population. Specifically, the ID population during pregnancy was chosen to ensure a more precise and safer intervention. Recent research has indicated that both high and low Hb levels can lead to negative prenatal and perinatal outcomes, highlighting the importance of avoiding excessive iron supplementation during pregnancy. Additionally, this study is pioneering in its investigation of iron supplementation in a pregnant population residing at Plateau areas.

However, this study also has some limitations. First, as a single-center retrospective cohort study, potential causal relationships cannot be drawn, and retrospective studies also have some inherent biases and missing data. Second, when exploring the effect of covariates on pregnancy outcomes, although the variables were adjusted, there may still be potential confounding factors that have not been addressed.

In future studies, we will design a prospective study to reduce bias and confounding, and conduct a more comprehensive study in multiple hospitals in the region to increase the generalizability of the results and the diversity of the sample. The follow-up period of the study was also increased to assess continued neonatal and maternal effects. Finally, we tried to include pregnant women in plateau areas from different regions to enhance the external validation and confirm the consistency and reliability of the results.

Finally, in this study, we found that SF < 30 μg/L as the standard of preventive iron supplementation could improve the effect of iron supplementation and reduce adverse maternal outcomes compared with Hb < 120 g/L as the standard of preventive iron supplementation. These findings suggest that it may be necessary to choose a more sensitive serum ferritin as the standard of prevention and treatment of iron deficiency during pregnancy in plateau area, so as to improve the pertinence and effectiveness of preventive iron supplementation during pregnancy and reduce adverse pregnancy outcomes caused by iron deficiency. At the same time, these findings can help colleagues to optimize the preventive measures of iron supplementation during pregnancy in plateau area and reduce the incidence of postpartum complications. It can provide valuable reference for pregnancy consultation and clinical treatment, and has important practical value and guiding significance.

## 5. Conclusions

In conclusion, compared with the ID pregnant women in plateau area who started iron prophylaxis with Hb < 120g/L as the standard, the preventive iron supplementation with SF < 30μg/L as the standard can reduce the incidence of ID and IDA before delivery, improve the recovery rate and recovery time of Hb level before delivery and SF ≥ 30μg/L, and reduce the incidence of postpartum hemorrhage, but does not reduce the incidence of other pregnancy complications and pregnancy outcomes such as: gestational hypertension, gestational diabetes, postpartum blood transfusion, neonatal asphyxia, premature birth, low birth weight infants. It is recommended to promote this prevention standard in qualified hospitals to improve the effect of iron supplementation in pregnant women with ID in early pregnancy and improve adverse pregnancy outcomes in plateau area.

## Acknowledgments

We express our gratitude to the patients and their families for their trust in our work. Additionally, we would like to acknowledge the Ethics Department and Science and Education Department of Kunming First People’s Hospital for their valuable oversight of this study. We would also like to thank Fengting MU, Master of Statistics, for her help with statistical testing in this paper. The assistance provided by the Data Collection Center of the Clinical Laboratory, Kunming First People’s Hospital, in collecting the data is greatly appreciated.

Supplemental digital content “Raw data” is available for this article (https://links.lww.com/MD/Q816).

## Author contributions

**Conceptualization**: Yang Yao, Lanxu Sun, Jing Luo.

**Data curation**: Yang Yao.

**Formal analysis**: Yang Yao.

**Funding acquisition**: Jing Luo, Zenglin Yang.

**Investigation**: Yang Yao, Lanxu Sun, Yan Yang.

**Methodology**: Yang Yao, Lanxu Sun, Xin Zuo, Zenglin Yang, Yan Yang, Jing Luo.

**Project administration**: Yang Yao.

**Writing – original draft**: Yang Yao.

**Writing – review & editing**: Yang Yao, Lanxu Sun, Xin Zuo, Zenglin Yang, Jing Luo.

## Supplementary Material
